# A functionally significant SNP in TP53 and breast cancer risk in African-American women

**DOI:** 10.1038/s41523-017-0007-9

**Published:** 2017-02-27

**Authors:** Maureen E. Murphy, Song Liu, Song Yao, Dezheng Huo, Qin Liu, Sonia C. Dolfi, Kim M. Hirshfield, Chi-Chen Hong, Qiang Hu, Andrew F. Olshan, Temidayo O. Ogundiran, Clement Adebamowo, Susan M. Domchek, Katherine L. Nathanson, Barbara Nemesure, Stefan Ambs, William J. Blot, Ye Feng, Esther M. John, Leslie Bernstein, Wei Zheng, Jennifer J. Hu, Regina G. Ziegler, Sarah Nyante, Sue A. Ingles, Michael F. Press, Sandra L. Deming, Jorge L. Rodriguez-Gil, Christopher A. Haiman, Olufunmilayo I. Olopade, Kathryn L. Lunetta, Julie R. Palmer, Christine B. Ambrosone

**Affiliations:** 1 Program in Molecular and Cellular Oncogenesis, The Wistar Institute, Philadelphia, PA 19104 USA; 2 Department of Biostatistics and Bioinformatics, Roswell Park Cancer Institute, Buffalo, NY 14263 USA; 3 Department of Cancer Prevention and Control, Roswell Park Cancer Institute, Buffalo, NY 14263 USA; 4 Department of Public Health Sciences, University of Chicago, Chicago, , IL 60637 USA; 5 Department of Medicine, Rutgers Robert Wood Johnson Medical School/Rutgers Cancer Institute of New Jersey, New Brunswick, , NJ 08903 USA; 6 University of North Carolina Lineberger Comprehensive Cancer Center, Chapel Hill, , NC 27514 USA; 7 Department of Surgery, College of Medicine, University of Ibadan, Ibadan, Nigeria; 8 Department of Epidemiology & Public Health, University of Maryland School of Medicine, Baltimore, MD 21201 USA; 9 Department of Medicine, Abramson Cancer Center, The Perelman School of Medicine at the University of Pennsylvania, Philadelphia, , PA 19104 USA; 10 Department of Family, Population and Preventive Medicine, Stony Brook University, Stony Brook, NY 11794 USA; 11 Laboratory of Human Carcinogenesis, National Cancer Institute, Bethesda, MD 20892 USA; 12 International Epidemiology Institute, Rockville, MD 20850 USA; 13 Vanderbilt University Medical Center, Nashville, , TN 37232 USA; 14 Department of Preventive Medicine, Keck School of Medicine and Norris Comprehensive Cancer Center, University of Southern California, Los Angeles, CA 90089 USA; 15 Cancer Prevention Institute of California, Fremont, CA 94305 USA; 16 Stanford University School of Medicine and Stanford Cancer Institute, Stanford, CA 94305 USA; 17 Division of Cancer Etiology, Department of Population Sciences, Beckman Research Institute, City of Hope, Duarte, CA 91010 USA; 18 Division of Epidemiology, Department of Medicine, Vanderbilt Epidemiology Center, and Vanderbilt-Ingram Cancer Center, Vanderbilt University School of Medicine, Nashville, TN 37232 USA; 19 Sylvester Comprehensive Cancer Center and Department of Public Health Sciences, University of Miami Miller School of Medicine, Miami, FL 33136 USA; 20 Epidemiology and Biostatistics Program, Division of Cancer Epidemiology and Genetics, National Cancer Institute, Bethesda, MD 20892 USA; 21 Department of Epidemiology, Gillings School of Global Public Health and Lineberger Comprehensive Cancer Center, University of North Carolina, Chapel Hill, NC 27514 USA; 22 Department of Pathology, Keck School of Medicine and Norris Comprehensive Cancer Center, University of Southern California, Los Angeles, CA 90089 USA; 23 Department of Medicine, University of Chicago, Chicago, , IL 60637 USA; 24 Department of Biostatistics, Boston University, Boston, , MA 02215 USA; 25 Slone Epidemiology Center at Boston University, Boston, , MA 02215 USA

## Abstract

A coding region polymorphism exists in the *TP53* gene (Pro47Ser; rs1800371) in individuals of African descent, which reduces p53 tumor suppressor function in a mouse model. It has been unclear whether this functionally significant polymorphism alters cancer risk in humans. This analysis included 6907 women with breast cancer and 7644 controls from the AMBER, ROOT, and AABC consortia. We used multivariable logistic regression to estimate associations between the TP53 Pro47Ser allele and overall breast cancer risk. Because polymorphisms in *TP53* tend to be associated with cancer risk in pre-menopausal women, we also limited our analyses to this population in the AMBER and ROOT consortia, where menopausal status was known, and conducted a fixed effects meta-analysis. In an analysis of all women in the AMBER, ROOT, and AABC consortia, we found no evidence for association of the Pro47Ser variant with breast cancer risk. However, when we restricted our analysis to only pre-menopausal women from the AMBER and ROOT consortia, there was a per allele odds ratio of 1.72 (95% confidence interval 1.08–2.76; *p*-value = 0.023). Although the Pro47Ser variant was not associated with overall breast cancer risk, it may increase risk among pre-menopausal women of African ancestry. Following up on more studies in human populations may better elucidate the role of this variant in breast cancer etiology. However, because of the low frequency of the polymorphism in women of African ancestry, its impact at a population level may be minimal.

## Genetics: Variant may increase cancer risk in pre-menopausal black women

A gene variant found in people of African descent may increase the risk of breast cancer—but only among pre-menopausal women. In a study of more than 14,000 women of African ancestry, a team led by Maureen Murphy from the Wistar Institute in Philadelphia, Pennsylvania, USA, and Christine Ambrosone from Roswell Park Cancer Institute in Buffalo, NY, USA, tested for an association between breast cancer and a rare genetic polymorphism in the TP53 gene that’s found almost exclusively in African-descent populations. This variant alters the p53 tumor suppressor protein and has been shown to increase cancer risk in a mouse model. Murphy and colleagues showed that the variant increased risk by about 72% in women who had not yet experienced menopause—which could help explain the earlier onset of the disease among women of African ancestry.

## Introduction

The p53 tumor suppressor (*TP53*) holds distinction as the most frequently mutated gene in human cancer. In addition to inactivating mutations in human cancer, there are a number of single-nucleotide polymorphisms (SNPs) in *TP53*, and in genes whose protein products regulate p53, *MDM2,* and *MDM4*; these SNPs modify TP53 (hereafter p53) pathway signaling, and can confer increased risk for cancer.^1–3^ Of note, the proline 72 allele in *TP53*, and the G allele of SNP309 in *MDM2*, have previously been associated with increased breast cancer risk in pre-menopausal women.^4, 5^ More than two decades ago, an inherited polymorphism in *TP53* that converts the proline residue at amino acid 47 to a serine was reported in African Americans (Pro47Ser, rs1800371, G/A).^6^ We showed that the serine 47 (hereafter S47) variant has significantly impaired ability to induce programmed cell death in multiple human cell lines engineered to contain inducible human forms of WT p53 and S47 (ref. 7). More recently, we created a mouse model for this variant, and showed that it confers significantly increased spontaneous cancer risk.^8^ The Pro47Ser polymorphism appears to be restricted to African-descent populations at a minor allele frequency (MAF) of 1.6%, and is extremely rare in populations of non-Finnish Europeans (3/126766), and non-existent in populations of Finnish Europeans (0/26082) or East Asian descent (0/18924), according to the recently released GnomAD data (http://gnomad.broadinstitute.org/variant/17-7579548-G-A). Because of the functional significance of this variant in cell lines and mouse models, we sought to test the hypothesis that the S47 variant might be associated with breast cancer risk in African American women, particularly among pre-menopausal women.

## Results

In the AMBER consortium, imputed data on the Pro47Ser polymorphism (rs1800371) were available for 3130 cases and 3698 controls. As shown in Table 1, the Pro47Ser SNP is rare, with a MAF of 1.4% in controls. The MAF of rs1800371 in all participating studies of the three consortia are shown in Supplementary Table 1, ranging from 0.9 to 1.8%. There was no association between rs1800371 and risk of overall breast cancer (odds ratio (OR) 1.17, 95% confidence interval (CI) 0.84–1.62, *p* = 0.34) (Table 1). Similarly, there were no significant associations observed in ROOT or in African American Breast Cancer Consortium (AABC).

**Table 1:** Associations between *TP53* rs1800371 and breast cancer risk in African-American women in the AMBER, ROOT, and AABC Consortia

*TP53* rs1800371	Controls	All cases	*p*-value	ER+cases	*p*-value	ER-cases	*p*-value
All women							
AMBER							
*N*	3698	3130	—	1674	—	963	—
MAF	0.014	0.016	—	0.015	—	0.016	—
OR (95% CI)*	—	1.17 (0.84–1.62)	0.34	1.08 (0.73–1.60)	0.70	1.18 (0.73–1.92)	0.49
ROOT							
N	2029	1657	—	403	—	374	—
MAF	0.011	0.012	—	0.017	—	0.013	—
OR (95% CI)**	—	1.10 (0.58–2.08)	0.78	2.31 (0.91–5.86)	0.077	1.04 (0.35–3.07)	0.95
AABC							
N	1917	2120	—	1117	—	591	—
MAF	0.014	0.014	—	0.013	—	0.014	—
OR (95% CI)	—	1.08 (0.71–1.65)	0.70	1.25 (0.74–2.12)	0.41	1.00 (0.53–1.86)	0.99
Premenopausal women							
AMBER							
N	1490	1218	—	631	—	398	—
MAF	0.013	0.019	—	0.016	—	0.019	—
OR (95% CI)*	—	1.79 (1.07–2.99)	0.027	1.52 (0.80–2.87)	0.20	1.98 (0.95–4.11)	0.07
ROOT							
N	898	724	—	146	—	169	—
MAF	0.010	0.012	—	0.015	—	0.013	—
OR (95% CI)**	—	1.39 (0.51–3.78)	0.52	1.84 (0.35–9.77)	0.48	1.17 (0.22–6.28)	0.86
Meta-analysis of AMBER and ROOT							
Per A allele OR (95% CI)***	—	1.72 (1.08–2.76)	—	1.64 (0.88–3.05)	—	1.72 (0.87–3.40)	—
*p*-value for testing association	—	0.023	—	0.12	—	0.12	—
*p*-value for testing heterogeneity	—	0.89	—	0.96	—	0.95	—

*Logistic regression models adjusted for age, principal components of ancestry, geographic region, study, and DNA source

**Logistic regression models adjusted for study sites, age, and principal components of the genotypes for ancestry

***Fixed effect meta-analysis based on study-specific ORs and SEs from BWHS, WCHS, CBCS, and ROOT

As noted above, previous studies of SNPs in p53 and MDM2 observed associations with breast cancer only among premenopausal women. Thus, we performed analysis among that group only. In both AMBER and ROOT, there were suggestions of increased risk of breast cancer with per allele ORs of 1.79 (95% CI, 1.07–2.99) and 1.39 (95% CI, 0.51–3.78), respectively. Because the SNP was imputed from genotype data in AMBER, we repeated this analysis limited to the >98% of pre-menopausal samples for which the maximum posterior genotype probability was >90%. The comparable OR was 2.07 (95% CI 1.18–3.65; *p* = 0.01).

**Fig. 1:**
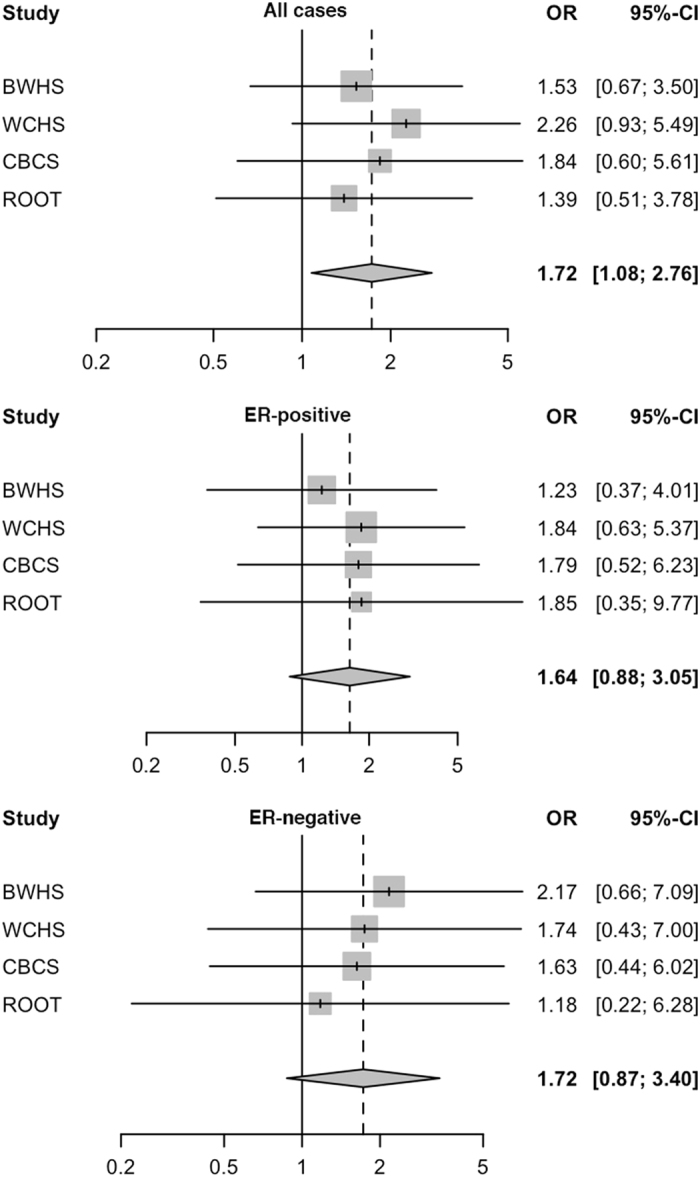
Forest plot of meta-analysis of *TP53* rs1800371 with breast cancer risk among pre-menopausal women in the AMBER and ROOT consortia. Study-specific odds ratios (OR) from each of BWHS, WCHS, CBCS, and ROOT is indicated as a *shaded square*, with 95% confidence interval (CI) shown as a *horizontal line*. The meta-OR is indicated as a *diamond*, with the width corresponding to the 95% CI. The analyses were performed for all cases, ER-positive cancer, and ER-negative cancer

In a fixed-effects meta-analysis of data from the ROOT and AMBER consortia where data of menopausal status were available, we observed a per-allele OR of 1.72, 95% CI, 1.08–2.76, *p* = 0.023 as shown in Table 1 and in a forest plot in Fig. 1.

## Discussion

We previously showed that, in human cell lines and a mouse model, the S47 variant exhibits a modest decrease in apoptosis in response to most genotoxic stresses compared with wild-type p53, but exhibits a significant defect in cell death induced by cisplatin.^8^ The variant shows impaired ability to transactivate a subset of p53 target genes, including two involved in metabolism: *Gls2* (glutaminase 2) and *Sco2*. We showed that mice expressing S47 in homozygous or heterozygous form are susceptible to spontaneous cancers of diverse histological types. Because of the strong functional significance of this variant, and it’s specificity to populations of African ancestry, it could play a role in the earlier onset and more aggressive nature of breast cancer among women of African descent.

Collaborating with three large consortia of breast cancer in women of African ancestry, our findings suggest that the S47 variant may contribute to increased risk of pre-menopausal breast cancer in individuals of African descent. However, because of the low prevalence of the variant, despite the fact that the studies are the largest available for breast cancer in women of African descent, associations were of borderline significance in AMBER and non-significant in ROOT. The combined meta-analysis, including 1942 pre-menopausal cases and 2388 controls, was also of borderline significance.

Although the suggestions of increased risk could be interpreted as null because of the borderline significance of the associations, the high frequency of sporadic cancer in the S47 mouse^8^ strengthens our hypothesis that this rare variant may increase risk among women of African ancestry, but the sample sizes do not allow for clear associations. The observed increased risk among premenopausal women from the meta-analysis might be driven by AMBER studies, which has a higher MAF than ROOT studies (1.5% vs. 1.1%). If admixture with European ancestry dilutes associations with this African-specific variant, which appeared to be more apparent in ROOT than in AMBER, that could explain the lack of significance in ROOT. It may thus need much larger studies to be able to observe a strong association of this rare SNP in African American populations, especially for testing heterogeneity by tumor estrogen receptor (ER) status. It is also possible that there truly is no effect of the S47 variant on human breast cancer. Even though the tumor suppressive function of the Pro47Ser protein is compromised in mice, the effect may be tissue specific, and not relevant in the mammary gland; similar findings have been found for other p53 variants.^9^ Nonetheless, studies such as these, taking findings from cell lines and mouse models to human populations, are needed to understand the basis of cancer etiology.

## Methods

Based upon our laboratory findings, we collaborated with investigators from the AMBER Consortium, which includes data and samples from the Black Women’s Health Study (BWHS), the Carolina Breast Cancer Study, and the Women’s Circle of Health Study (WCHS).^10^ All studies were approved by the affiliated institutional review boards. BWHS is a prospective cohort study with participants across the US enrolled by mailed questionnaires and followed with biennial and 5-year interval follow-up questionnaires. CBCS and WCHS are both case–control studies, with CBCS 1 and 2 conducted with population-based sampling and in-person interviews from 1993–2001 in 24 counties in North Carolina. WCHS, initiated in 2002 in metropolitan New York City and several counties in eastern New Jersey, is still ongoing in NJ. For these analyses, we included samples from women with invasive cancer or ductal carcinoma in situ (*n* = 3130), confirmed by pathology reports or registry records from which we also obtained data on ER status, and 3698 controls. Genotyping was performed at the Center for Inherited Disease Research as part of a larger project, using the Illumina HumanExome Beadchip Plus v1.1 plus 200,000 custom beadtypes. Imputation based on 1000 Genomes data was carried out at the University of Washington. The observed:expected variance ratio, a measure of squared correlation between the imputed genotypes and the true genotypes for the imputed SNP was 0.91. We examined associations between the imputed SNP rs1800371 in TP53 and breast cancer overall, ER positive cancer, and ER negative disease. Because previous studies of SNPs in *TP53* and *MDM2* have found associations only in pre-menopausal women,^4, 5^ we also limited analyses to only that group. The odds ratios and 95% confidence intervals (CIs) were derived from multivariable logistic regression models, which adjusted for study (BWHS, WCHS, or CBCS), age (in 10 year groupings), DNA source (blood, saliva, mouthwash) and geographic location (New Jersey, Northeast minus New Jersey, South, Midwest or West). Because ancestry is a recognized confounder of genetic associations, principal components of the genotypes for ancestry with *p* < 0.1 after including the covariates listed above in the model were also included. We genotyped twenty samples in a blinded manner using Taqman analysis and primers from Applied Biosystems for the rs1800371 SNP in the Wistar Institute Genomics Facility; all Taqman genotyping was 100% concordant with imputed data. All analyses were conducted using SAS 9.4 (SAS Institute, Cary, NC).

Because of the relatively low frequency of the S47 SNP, we sought to expand our analysis by collaborating with two additional consortia. The ROOT consortium includes 1657 breast cancer cases and 2029 controls from six studies in African American, African Barbadian, and African women, including the Nigerian Breast Cancer Study, the Barbados National Cancer Study, the Racial Variability in Genotypic Determinants of Breast Cancer Risk Study, the Baltimore Breast Cancer Study, the Chicago Cancer Prone Study, and the Southern Community Cohort.^11, 12^ All studies were approved by the affiliated institutional review boards. Genotyping was performed using the Illumina HumanOmni2.5-8v1 array as part of a genome-wide association study.

AABC includes cases and controls from the following epidemiologic studies: The Multiethnic Cohort study, 734/1003; The Los Angeles component of The Women’s Contraceptive and Reproductive Experiences (CARE) Study, 380/224; The San Francisco Bay Area Breast Cancer Study, 172/23; The Northern California site of the Breast Cancer Family Registry, 440/53; The Nashville Breast Health Study, 310/186; and The Wake Forest University Breast Cancer Study, 125/153). The present analysis includes AABC GWAS data for 2225 AA women with invasive breast cancer and 1983 controls.^13^ Although WCHS and CBCS participated in AABC, samples from those studies were included as part of the AMBER consortium described above.

We also performed a fixed effects meta-analysis of risk of pre-menopausal breast cancer using data from AMBER and ROOT. Because the sample size of some participating studies in ROOT was small, especially after stratification by menopausal status and ER status, the study-specific estimates for this rare SNP were not stable. Therefore, in the meta-analysis we used risk estimates from ROOT as one study but study-specific risk estimates from BWHS, WCHS, and CBCS, where adequate sample size and reliable estimates were available from each study. AABC was excluded from these analyses because menopausal status was not known for their participants.

## Electronic supplementary material

Supplementary Information
