# Supplementary material for: A functionally significant SNP in TP53 and breast cancer risk in African-American women
Source: NPJ Breast Cancer. 2017 Feb 27;3:5. doi: 10.1038/s41523-017-0007-9 (PMC5445618; doi:10.1038/s41523-017-0007-9)
Supplement: Supplementary file 1 — Supplementary Information [file 41523_2017_7_MOESM1_ESM.docx]

**Supplementary Table 1. Minor allele frequency of TP53 rs1800371 by studies in AMBER and ROOT consortia**

| **Study** | **MAF** |
| --- | --- |
| **AMBER** | **0.015** |
| BWHS | 0.014 |
| WCHS | 0.015 |
| CBCS | 0.015 |
| **ROOT** | **0.011** |
| BNCS | 0.009 |
| BBCS | 0.010 |
| NBCS | 0.010 |
| CCPS | 0.014 |
| RVGBC | 0.010 |
| SCCS | 0.014 |
| **AABC** | **0.014** |
| WFBC | 0.010 |
| BCFR | 0.018 |
| CARE | 0.014 |
| MEC | 0.013 |
| PLCO | 0.009 |
| SFBC | 0.013 |
| NBHS | 0.017 |
